# Glial cells, but not neurons, exhibit a controllable response to a localized inflammatory microenvironment *in vitro*

**DOI:** 10.3389/fneng.2014.00041

**Published:** 2014-11-14

**Authors:** Salah Sommakia, Jenna L. Rickus, Kevin J. Otto

**Affiliations:** ^1^Weldon School of Biomedical Engineering, Purdue UniversityWest Lafayette, IN, USA; ^2^Physiological Sensing Facility at the Bindley Bioscience Center and Birck Nanotechnology Center, Purdue UniversityWest Lafayette, IN, USA; ^3^Department of Agricultural and Biological Engineering, Purdue UniversityWest Lafayette, IN, USA; ^4^Department of Biological Sciences, Purdue UniversityWest Lafayette, IN, USA; ^5^J. Crayton Pruitt Family Department of Biomedical Engineering, University of FloridaGainesville, FL, USA

**Keywords:** intracortical microelectrodes, foreign body response, primary cortical cultures

## Abstract

The ability to design long-lasting intracortical implants hinges on understanding the factors leading to the loss of neuronal density and the formation of the glial scar. In this study, we modify a common *in vitro* mixed cortical culture model using lipopolysaccharide (LPS) to examine the responses of microglia, astrocytes, and neurons to microwire segments. We also use dip-coated polyethylene glycol (PEG), which we have previously shown can modulate impedance changes to neural microelectrodes, to control the cellular responses. We find that microglia, as expected, exhibit an elevated response to LPS-coated microwire for distances of up to 150 μm, and that this elevated response can be mitigated by co-depositing PEG with LPS. Astrocytes exhibit a more complex, distance-dependent response, whereas neurons do not appear to be affected by the type or magnitude of glial response within this *in vitro* model. The discrepancy between our *in vitro* responses and typically observed *in vivo* responses suggest the importance of using a systems approach to understand the responses of the various brain cell types in a chronic *in vivo* setting, as well as the necessity of studying the roles of cell types not native to the brain. Our results further indicate that the loss of neuronal density observed *in vivo* is not a necessary consequence of elevated glial activation.

## Introduction

Implantable intracortical microelectrodes hold great potential as neural prostheses for the treatment of a wide range of traumatic and degenerative injuries to the central nervous system, but suffer from unreliability in chronic settings. This decline in chronic device performance correlates with a reactive response of brain tissue (Vetter et al., 2004). Designing therapeutic approaches to counter this decline in device performance is complicated by the lack of detailed mechanistic understanding of the progression of the reactive tissue. Dural and vascular damage appear to be major factors contributing to the reactive tissue response (Karumbaiah et al., 2013; Saxena et al., 2013). Using novel device capture techniques (Woolley et al., 2011, 2013a,b), this reactive tissue response has been shown to be non-uniform and depth dependent, with stronger scarring closer to the surface of the brain (Woolley et al., 2013c). Transdural implants elicit a much greater response than implants dwelling completely within the brain (Markwardt et al., 2013). These findings collectively suggest that the introduction of non-native cellular and molecular components into the brain amplifies inflammatory pathway activation, and that this activation is strongest at the site of injury to respective structures. Recently, potential therapeutic targets such as reactive oxygen species and toll-like receptor 4 (TL4) have been identified (Potter et al., 2013; Ravikumar et al., 2014), but the complexity underlying *in vivo* conditions can obscure investigations of biological mechanisms.

These obstacles can be somewhat overcome by studying simpler models, such as *in vitro* cell cultures. The most widely used model, first described by Polikov et al. (2006, 2009), presents microscale foreign bodies to primary mixed neural cultures., and has been applied to test biocompatibility of various materials as neural interfaces (Achyuta et al., 2010; Tien et al., 2013). This model requires the modification of the culture media to achieve a globally elevated activation state. We posit that a more localized inflammatory microenvironment may better represent the non-uniform reactive tissue response, and propose a modification to the model whereby the foreign objects are dip-coated in lipopolysaccharide (LPS) to simulate a localized inflammatory microenvironment. LPS is a known upregulator of microglial activation through TL4 binding (Lehnardt et al., 2003; Tzeng et al., 2005), and as such is an attractive option for modifying the Polikov model to test cellular responses to localized targeting of TL4 receptors. In contrast to the previous model, the creation of a localized inflammatory microenvironment also enables the analysis of neuronal responses.

Previous research in the neurotrauma field has also found that, due its surfactant properties, soluble polyethylene glycol (PEG) can induce membrane sealing of damaged cells and reduce edema (Borgens et al., 2002). This effect significantly improves recovery from both spinal cord and traumatic brain injuries by inducing cellular and behavioral recovery (Koob et al., 2005, 2008; Koob and Borgens, 2006). Additionally, we have recently showed that a dip-coated PEG film can modulate impedance changes caused by non-cellular components both *in vitro* and *in vivo* (Sommakia et al., 2014). In this regard, a non-grafted dip-coated PEG film is a technically and economically attractive option to achieve both antifouling and membrane sealing. Our hypothesis is that a dip-coated layer of high molecular weight PEG will exhibit sufficient short term stability to modulate cellular responses to microelectrodes *in vitro*. Given the importance of the early stages of the injury response in shaping the later chronic stages, this approach might prove highly beneficial *in vivo*.

In this work we test our PEG hypothesis using the local inflammation-modified Polikov model. We show that, as expected, coating segments of microwire with LPS results in an increase in microglial activation at distances up to 150 μm, and, importantly, co-depositing LPS with a PEG solution prevents observed increases in microglial activation. We also observe a slight increase in astrocyte activation in response to LPS-coated microwire, but not at the same magnitude or spatial distribution as microglia. Interestingly, neuronal responses in this *in vitro* paradigm do not appear to be influenced by corresponding glial responses.

## Materials and methods

### Cell culture and microwire placement

The experimental procedures complied with the Guide for the Care and Use of Laboratory Animals and were approved by The Purdue Animal Care and Use Committee (PACUC). Forebrains from E17 embryonic rat pups were received suspended in 5 ml of Solution 1 (NaCl 7.24 g/L; KCl 0.4 g/L; NaH2PO4 0.14 g/L; Glucose 2.61 g/L; HEPES 5.96 g/L; MgSO4 0.295 g/L; Bovine Serum Albumin 3 g/L) in a 50 ml centrifuge tube. Under sterile conditions, the tissue was gently triturated with an added 18 μl of trypsin solution (Sigma-Aldrich, St. Louis, MO) (7.5 mg/ml in 0.9% saline) and incubated for 20 min in a 37°C water bath. Following the incubation step, 100 μl of trypsin inhibitor/DNAase solution (Sigma-Aldrich, St. Louis, MO) (2.5 mg/ml trypsin inhibitor, 400 μg/ml DNAase in 0.9% saline) was added and tissue was again gently triturated. The tissue was then centrifuged at 1,000 rpm for 5 min at room temperature and supernatant was poured off. Cells were re-suspended in 16 ml of Hibernate-E (Brainbits, Springfield, IL) and triturated once again. Cells were filtered through a 70 μm cell strainer (Fisher Scientific) and centrifuged at 1,400 rpm for 5 min at room temperature. Supernatant was poured off and cells were re-suspended in a culture medium consisting of Dulbecco’s modified Eagle’s Medium (DMEM) with 10% Fetal Bovine Serum (FBS) and 10% horse serum (HS). The cells were then seeded in 96 well plates at a density of 500,000 cells/cm^2^, and cultured for 7 days at 37°C and 5% CO_2_, with the cell media being replaced every 48 h. At day 7 *in vitro*, lengths of 50 μm-diameter tungsten microwire (California Fine Wire Co., Grover Beach, CA) were autoclaved then cut into small segments of 5–7 mm in length using carbide scissors. The microwire segments were treated by dip coating with one of four treatments: LPS (50 ng/ml) only, PEG (20% aqueous solution, 4000 MW) only, a 1:1 mixture of LPS and PEG, or uncoated. A relatively low LPS concentration was chosen based on reported literature values (Das et al., 1995; Wang et al., 2005) in order to achieve localized activation of microglia, but prevent a generalized activation that might result from a higher concentration of LPS diffusing rapidly throughout the well. PEG concentration is based on our previous work demonstrating a proof of concept for using PEG to modulate impedance changes to neural microelectrodes (Sommakia et al., 2014). In each well, one segment of microwire was dropped into the medium and allowed to sink to the bottom of the well. The plates were then placed in the incubator for an additional 7 days.

### Cell fixing and labeling

At day 14 *in vitro*, the cultures were fixed with 4% paraformaldehyde for 10 min, rinsed 3× with HEPES Buffered Hank’s saline (HBHS) (in g/L; 7.5 g NaCl, 0.3 g KCl, 0.06 g KH2PO4, 0.13 g Na2HPO4, 2 g Glucose, 2.4 g HEPES, 0.05 g MgCl2:6H2O, 0.05 g MgSO4:7H2O, 0.165 g CaCl2, 90 mg NaN3, at pH 7.4), then permeabilized with 0.2% Triton-X (Sigma-Aldrich, St. Louis, MO). The cultures were then blocked with 10% normal goat serum (Jackson Immunoresearch, West Grove, PA) for 1 h, after which primary antibodies to beta-3-tubulin (β-3-tub) (Covance, Princeton, NJ), which labels neurons; Glial Fibrillary Acidic Protein (GFAP) (Millipore, Billerica, MA), which labels astrocytes; and Ionized Calcium binding adaptor molecule 1 (Iba1) (Wako, Osaka, Japan), which labels microglia, were added, and the cultures incubated in a 4°C refrigerator overnight. The wells were then aspirated, rinsed in HBHS 3×, and the following secondary antibodies were added: Alexa Fluor 488 Goat anti-mouse, Alexa Fluor 555 Goat anti-chicken, and Alexa Fluor 635 Goat anti-rabbit (Invitrogen, Carlsbad, CA). After a 2 h incubation at room temperature, the secondary antibodies were rinsed 3× with HBHS, and a final volume of 100 μl of HBHS was left in the wells for imaging. Special care was taken to ensure the microwire segments remained attached to the bottom of the wells.

### Image acquisition and analysis

**Figure 1:**
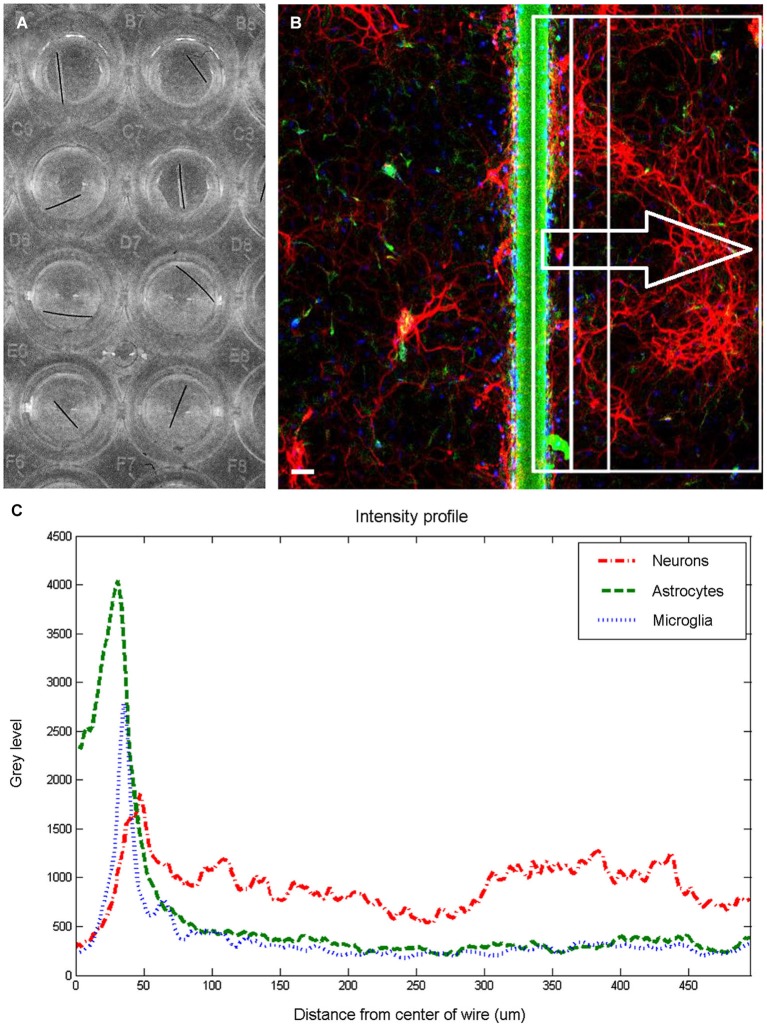
**Image quantification**. Wells in 96 well plate **(A)** were imaged to produce a fluorescent image **(B)** and extract intensity profiles for each channel. The fluorescent image is pseudocolored to show neurons in red, astrocytes in green, and microglia in blue. Scale bar is 50 μm. For each examined region (examples shown within rectangles), three intensity profiles **(C)** are generated, and response indices calculated by summing the area under the graph corresponding to the chosen distances and dividing by 10000.

Fluorescent images (512 × 512 pixels) were obtained on a confocal microscope fitted with a long working distance 10× air objective using Fluoview software (Olympus, Center Valley, PA). The different channels were imaged sequentially, and noise reduction was achieved by applying a Kalman filter built into the acquisition software to 3 scans for each channel. Each plate was imaged using the same set of imaging parameters (laser power, aperture, acquisition time) to ensure uniformity. Source images were imported into ImageJ (ImageJ, U. S. National Institutes of Health, Bethesda, MD), visually inspected and rotated to place the microwire in a vertical orientation. When possible, two adjacent rectangular selections, 480 pixels high by 240 pixels wide (equivalent to 994 μm by 496 μm), were made with the long edge running on the center of the wire. If that was not possible due to excessive proximity to wall of the well, only a single rectangular selection was made facing the interior of the well. Each of these selections was considered a single sample for analysis purposes. From these selections, intensity profiles of average brightness of each vertical line were generated, as shown in Figure 1C. Microwire segments were also imaged in three empty wells, and an average intensity profile was obtained and subtracted from the intensity profile generated from cell-containing wells. One response index (RI) per cell type was obtained for each region by summing the area underneath the intensity profile line between the distance points corresponding to the region boundaries and dividing by 10000. Statistical analysis was performed using the SAS 9.3 statistical package (SAS Institute Inc., Cary, NC). A general linear model (GLM) procedure was used perform to a one way ANOVA with block, to remove the effects of variations between the plates by treating the plates as a statistical block. *Post hoc* Tukey tests were used to determine statistical significance between the treatment groups at a significance level of *α* = 0.05. The error bars plotted represent the standard error of the means. *P*-values less than 0.05 are denoted in the figures by a single asterisk, while *p*-values less than 0.001 are denoted by double asterisks. Plots were generated using MATLAB (The MathWorks Inc., Natick, MA).

## Results

Figure 1 shows an overview of the methodology employed to analyze the cellular responses to microwire segments. Microwire segments placed in the wells (Figure 1A) were imaged, resulting in sets of images such as the one shown in Figure 1B. Intensity profiles (Figure 1C) of areas of various widths were analyzed to obtain the results described below.

### Microglia

**Figure 2:**
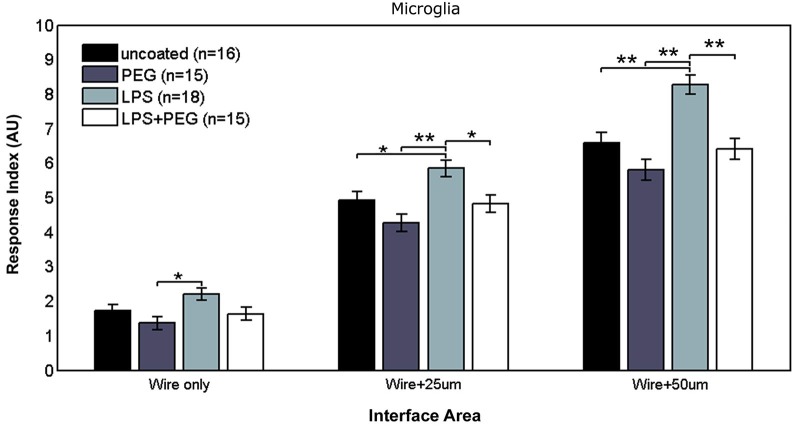
**LPS-coated microwire elicits higher microglial response in interface areas, and co-deposition of PEG with LPS reduces microglial response to control levels**.

Figure 2 shows the different levels of aggregate microglial response in interface areas of different sizes. In the interface area containing only the microwire (i.e., 25 μm), the only significant difference in the microglial RI was between the PEG coated microwire and LPS coated microwire (RI = 1.37 vs. 2.2, *p* = 0.007). For the interface area containing the wire and extending over an adjacent 25 μm, significant pairwise differences were observed between the LPS coated wire (RI = 5.84) and the other wires (uncoated RI = 4.92, *p* = 0.041; PEG RI = 4.26, *p* < 0.0001; LPS + PEG RI = 4.82, *p* = 0.022). For a wider interface area containing the microwire and extending over the adjacent 50 μm, these pairwise differences get stronger between the LPS coated wire (RI = 8.27) and the other wires (uncoated RI = 6.58, *p* = 0.0007; PEG RI = 5.8, *p* < 0.0001; LPS + PEG RI = 6.4, *p* = 0.0002). Notably, the relative pattern of the microglia response indices for the different conditions is the same for all three regions. Only the overall magnitude of the responses increase as the anti-Iba1 fluorescence is summed over larger areas. This indicates that all three size regions up to the wire plus 50 microns are representative of interface. In all three cases LPS induced microglia activation rises to a level statistical significance. As more distance is included in the interface measurement, the ability of the PEG to relieve microglia activation by LPS rises to statistical significance.

**Figure 3:**
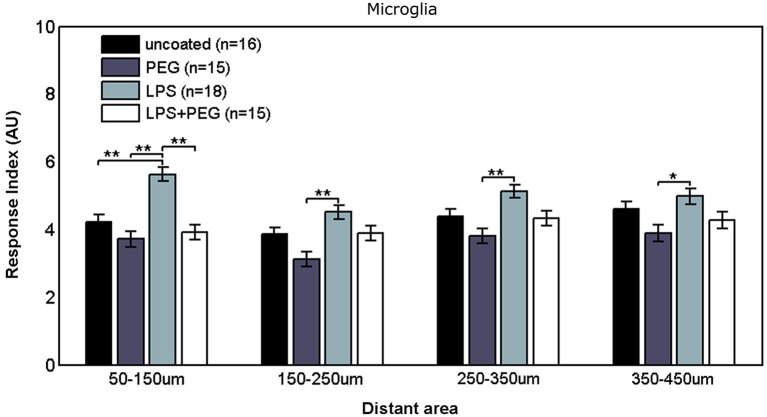
**Microglial response to LPS-coated microwire compared to all other treatments is elevated at distances up to 150 μm, after which a tiered response is observed**.

Figure 3 shows the microglial responses at more distant regions. For the closest distant bin extending from 50 to 150 μm from edge of wire, the RI for LPS coated wire (RI = 5.62) was significantly higher than all the other treatments (uncoated RI = 4.21, *p* = 0.0001; PEG RI = 3.71, *p* < 0.0001; LPS + PEG RI = 3.91, *p* < 0.0001). For the next three distant 100 μm wide bins, the only significant difference observed was between LPS coated wire and PEG coated wire in all 3 bins. These calculated RI are as follows: for the bin extending from 150 to 250 μm from edge of wire: LPS RI = 4.5 vs. PEG 3.12, *p* = 0.0001; for the bin extending 250–350 μm from edge of wire: RI = 5.12 vs. 3.8, *p* = 0.0003; for the bin extending 350–450 μm from edge of wire): RI = 4.98 vs. 3.9, *p* = 0.01. Again the pattern of relative RI’s is consistent at all distances in the distant regions and matches that of the interface region. In this case the size of each measurement area is the same across cases and is always 100 microns. Again LPS induces an increased RI in all regions reaching statistical significance when comparing LPS to the PEG coated wire and in the most near distant region (50–150 microns) when comparing LPS to an uncoated wire. The wire likely induces some basal level of microglial attachment or activation, which is reduced by PEG alone, therefore the effect of LPS is most pronounced when we compare the PEG wire. These results are consistent with a diffusion based model whereby the effect of LPS will decrease with increasing distance from the wire.

### Astrocytes

**Figure 4:**
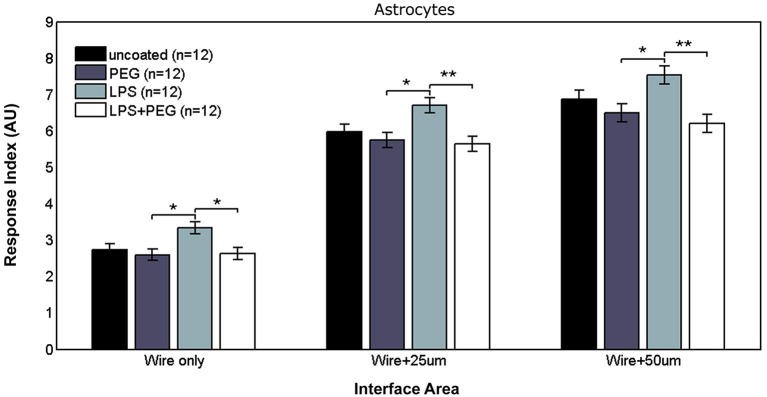
**Astrocytes in interface areas of varying width exhibit a tiered response to microwires coated with PEG, with or without LPS**.

Figure 4 shows the astrocyte RI at interface areas. For the interface area containing only the microwire, the astrocyte RI for LPS coated wire (RI = 3.33) was significantly higher than PEG coated and LPS + PEG coated wire (PEG RI = 2.59, *p* = 0.015; LPS + PEG RI = 2.63, *p* = 0.02). For the interface area containing the wire and extending an adjacent 25 μm, the same pairwise difference were observed, but with a stronger difference between the LPS coated wire (RI = 6.7) and the LPS + PEG coated wire (PEG RI = 5.75, *p* = 0.012; LPS + PEG RI = 5.64, *p* = 0.0045). For the interface area containing the microwire and extending an adjacent 50 μm, the same observation of the LPS astrocyte RI being higher than both PEG and LPS + PEG was noticed (LPS RI = 7.54, PEG RI = 6.49, *p* = 0.02; LPS + PEG RI = 6.19, *p* = 0.002). Overall the astrocytes show a similar pattern in the interface as the microglia, but to a lesser extent. Importantly, for all three interface sizes (at the wire, within 25 μm of the wire, and within 50 μm of the wire), the PEG coating is able to significantly reduce the LPS-induced astrocyte response.

**Figure 5:**
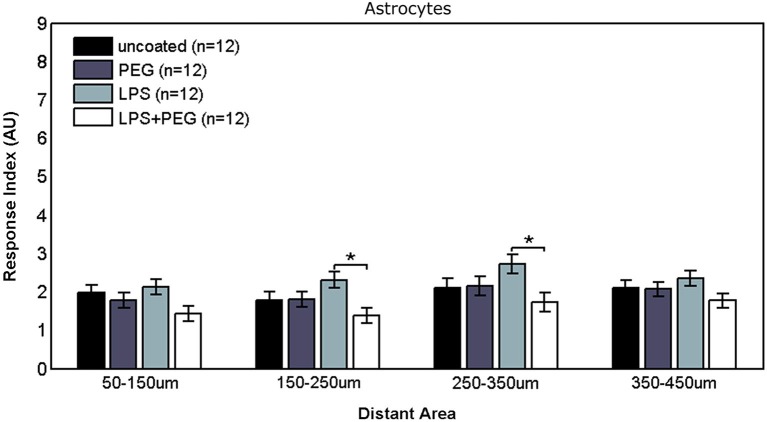
**Differences in astrocyte responses in distant areas appear between LPS and LPS + PEG coated microwires at the middle of the distance range analyzed**.

Figure 5 shows the astrocyte RI at distant areas. No significant differences were observed between the different treatments for the closest distant bin extending from 50 to 150 μm from edge of microwire. For the middle two distant bins, a slightly significant difference was observed between LPS coated wire and LPS + PEG coated wire [bin 2 (150–250 μm from edge of wire): LPS RI = 2.31, LPS + PEG RI = 1.37, *p* = 0.012; bin 3 (250–350 μm from edge of wire): LPS RI = 2.73, LPS + PEG RI = 1.73, *p* = 0.03].

### Neurons

**Figure 6:**
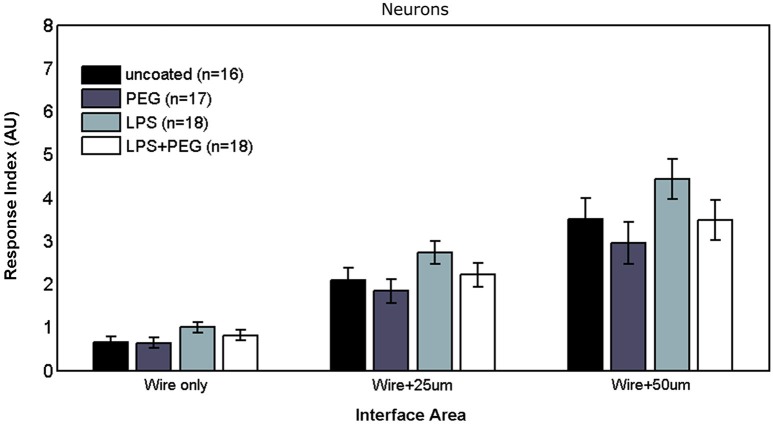
**No differences are observed in neuronal responses in interface areas of various widths**.

**Figure 7:**
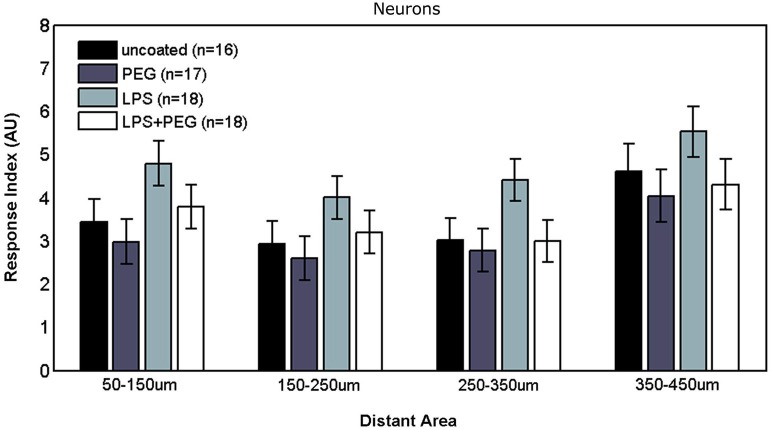
**No differences are observed in neuronal responses in distant areas**.

Figures 6, 7 show the neuron RI in interface and distant regions respectively. No significant differences in the neuron response were found between any of the treatment conditions in either interface or distant region. In contrast to microglia and astrocytes, where the RI was higher in distant areas in comparison to the widest interface area examined, the neuron RI in distant areas was roughly equal to that in the widest interface area examined.

## Discussion

### Validity of model system

To test the effects of a dip coated PEG film on the cellular responses to implanted electrodes, we modified a robust and frequently replicated *in vitro* mixed cortical culture model pioneered by Polikov et al. (2006, 2009, 2010; Achyuta et al., 2010; Tien et al., 2013). The anatomical and physiological complexity *in vivo* obscures mechanistic investigations into the reactive tissue response to implanted microelectrodes. Using an *in vitro* model allows us to simplify the biological system under study, and isolate particular components of interest. The challenge with *in vitro* models is to simulate physiological conditions in the absence of particular anatomical structures. In this particular model of primary cortical cell cultures, the cells exist in isolation from supporting vasculature, structural extra-cellular matrix components, and meninges. These aforementioned structures are heavily damaged during microelectrode insertion, which has been shown to strongly affect the chronic response of the brain to implanted microelectrodes (Karumbaiah et al., 2013; Markwardt et al., 2013; Saxena et al., 2013). The original model (Polikov et al., 2006) did not elicit a consistent glial scar, and it was necessary to alter the composition of the culture media to place all glial cells in the culture in an elevated reactive state, thereby ensuring a consistent glial scar (Polikov et al., 2009). By coating LPS directly onto microwire, we are able to create a localized inflammatory microenvironment that more closely mimics the reality of an indwelling cortical implant, rather than placing the glial cells in the culture in a globally activated state. This localized inflammatory microenvironment enables us to examine distance related effects on the cultured cells. For the LPS + PEG condition, concerns about cross contamination and the potential to disrupt the dip-coated PEG film led to the decision to co-deposit PEG and LPS via dip-coating from a single pot. While polymeric films containing PEG have the potential for prolonged drug release, they are typically crosslinked to form hydrogels (Peppas, 1997; Lin and Anseth, 2009) or composites (Ramakrishna et al., 2001). Dip-coated films of a pure hydrophilic polymer, such as PEG, are rarely used for prolonged drug release due to their burst release characteristics and potential for dissolution over timescales shorter than is therapeutically beneficial (Acharya and Park, 2006). PEG, in various conformations, has been shown to accelerate the release of small hydrophobic molecules similar to LPS (Ooya et al., 2003; Kang et al., 2007). For these aforementioned reasons, we were confident that our codeposition of PEG and LPS would not hinder the exposure of the cells to LPS.

To examine microglial response, we chose to quantify Iba1 fluorescence across relatively wide bins. The choice of Iba1 was due to its high specificity to the microglia/macrophage cell type. The function and level of Iba1 expression is directly related to the classic morphological changes associated with microglial activation (Ito et al., 1998). Iba1 crosslinks actin and is involved in the formation of membrane ruffles and rapid motility (Sasaki et al., 2001). Additionally, Iba1 levels correlate directly with morphological feature changes associated with microglial activation (Kozlowski and Weimer, 2012). While current morphological analysis methods have been found lacking (Beynon and Walker, 2012), the method of quantification we employ is not without its shortcomings. Given our bin sizing, an increase in fluorescence in a bin could be attributed to either an increase in microglial cell numbers, or a higher level of Iba1 expression, or both. However, proliferation, migration, and morphological changes are all important components of microglial activation. Quantification of Iba1 fluorescence in a given area can therefore capture an aggregate of these aspects of microglial activation, but cannot distinguish between the individual components. We chose our method of quantification of Iba1fluorescence using bin sizes of up to 100 μm as an indicator of microglial response because we were most interested in quantifying gross activation across an extended distance from the foreign body. This resulted in a tradeoff against smaller bin sizes and higher magnification examination of individual microglia. Similar image analysis approaches quantifying fluorescence levels have been used *in vitro* (Polikov et al., 2009, 2010; Achyuta et al., 2010; Tien et al., 2013) and *in vivo* (Azemi et al., 2011; Potter et al., 2013, 2014) to analyze responses to microelectrodes and microscale foreign bodies., while presenting similar shortcomings in terms of elucidating separate aspects of microglial activation. Additional markers of microglial activation, such as secreted cytokines, are also a major factor of interest when studying microglial responses. Commercially available biochemical assays are not sensitive enough to detect secreted cytokines in this particular *in vitro* injury model. Future studies should examine improved experimental and analysis methodologies to combine gross microglial responses with morphological changes and biochemical expression patterns.

### Analysis of cellular responses

#### Microglia

The microglial response in a narrow interface region comprising only the area under the microwire exhibits a three tiered response where a significant difference exists between the LPS only and the PEG only treatments, but not between the other conditions. This tiered response might be attributed to the difference between increased activation caused by the LPS and reduced cellular adhesion caused by PEG. The three data sets from the interfacial region included in Figure 2 (wire only, wire + 25 μm, wire +50 μm) examine the RI of the microglia near the wire by summing the fluorescence over progressively increasing areas. We note that all three sets have the same relative trend when we compare each condition (bare wire, PEG only, LPS, LPS + PEG), only the magnitudes increase as the sets progress because the summation area increases. We observe a microglial monolayer forming at the surface of the wire, explaining the lack of a significant difference between the different treatments. The difference becomes significant only at the wire +50 μm because the magnitudes of the fluorescence become large enough to detect the differences given the variance of the assay. Even though this effect is not statistically significant when we analyze the wire only and wire +25 μm, the trend is consistent. The same is true for LPS vs. a bare wire, although this effect becomes significant at both wire +25 μm and wire +50 μm. We therefore think that the effect of LPS to locally activate microglia and mitigate that activation by a PEG coating is happening throughout the entire interfacial area. This increase in microglial response might be explained by elevated activation of microglia through amplification of inflammatory pathways precipitated by TL4 binding, leading to an increase in microglial response at distance. The observed elevation of Iba1 fluorescence persists in the next 100 μm wide distant region, again indicating an extended inflammatory response, potentially mediated by secreted cytokines produced by activated microglia but dissipates in further distant regions, reverting to a tiered response, where the only significant pairwise difference is between LPS and PEG. This tiered response can again be attributed to distinct pathway amplification between the two treatments; the difference appearing only between the increased upregulation of microglial activation due to LPS and the reduced microglial activation due to PEG.

#### Astrocytes

In interface regions of varying width, the astrocyte response also exhibits a three tiered response, where an elevated astrocyte response is observed with LPS, and a reduction occurs with both PEG conditions (with LPS and without). In the distant regions, the first and fourth 100 μm wide distant bin do not exhibit any differences between the different treatments, but we observe a difference between LPS and LPS + PEG in the middle two 100 μm wide bins, but surprisingly no difference between LPS and PEG in these distant areas. A potential explanation is that the astrocytes are exhibiting a dose dependent response to LPS. Under this explanation, the increased activation in the interface area for the LPS only treatment results in both astrocyte migration from distant regions and increased overall proliferation; delivering the LPS with PEG results in astrocyte migration without an accompanying equivalent increase in proliferation, resulting in a depletion of distant astrocytes; while PEG only results in even less astrocyte activation in interface areas, which in turn does not signal migration of distant astrocytes. Because we did not directly test for whether the LPS was acting through direct binding to receptors on astrocyte surfaces, we are merely discussing correlative effects. It is unclear whether the astrocyte response is due to direct action by LPS, or if it they are reacting to cytokines and chemokines secreted by microglia. While astrocytes are not typically thought to express TL4 receptors, there is some evidence to the contrary (Bowman et al., 2003). Additionally, while GFAP-positive astrocytes are observed in primary cultures, a considerable portion of them differentiate *in vitro* from astrocyte precursors (Abney et al., 1981). It is possible that due to these culture conditions that astrocyte response is altered from normally developing astrocytes *in vivo*.

#### Neurons

No significant differences were detected in neuron response to any of the treatments, in either interface or distant regions. While not statistically significant, a coupling between neuron and astrocyte response can be noticed, where slightly higher (but not significantly different) neuron growth was observed for the LPS treatment. Neuronal growth has been consistently shown to occur on a supporting substrate of astrocytes (Noble et al., 1984; Tomaselli et al., 1988). In contrast to the microglia and astrocytes, where the response in the widest interface bin was considerably higher than the first adjacent distant bin, the neuron RI in the first distant bin was comparable to the neuron RI in the wide interface bin, and we did not observe a decline in neuron density over distance. One explanation mirrors the concern expressed earlier about the maturity of the astrocytes, where immature astrocytes in culture provided a better substrate for neuron outgrowth compared to mature astrocytes (Smith et al., 1990). An alternative explanation is that elevated glial activation is not in and of itself neurotoxic or neurodegenerative within a foreign body reactive tissue response paradigm. If the latter explanation is correct, then the loss of neural density *in vivo* following implantation of a microelectrode might be better explained by displacement of neurons following insertion trauma and edema which fail to reoccupy depleted zones because of the glial scar formation, or that *in vivo* neurotoxicity occurs due to direct contact between neurons and extrabrain components.

## Conclusions

We have shown that microglial response in a primary mixed cortical culture can be manipulated by dip-coated treatments. Microglial response can be increased by coating the surface of the foreign body with LPS, and this increase can be prevented by co-depositing LPS and PEG. We hypothesize that the film of high molecular weight PEG, while allowing for LPS release, presents a hydrated physical barrier that disrupts cytokine, chemokine and adsorbed protein gradients that typically guide pathological responses. Astrocyte response also increased for LPS coated foreign bodies, but it is unclear whether this response is directly mediated by LPS or whether it is caused by other microglia-secreted factors. Neuron response was not negatively correlated with microglial response, suggesting mechanisms other than glial activation causing *in vivo* neuronal density loss. Our results highlight the importance of considering the *in vivo* chronic foreign body response as a complex phenomenon with multiple, interconnected yet parallel processes. Attempts to target an individual brain cell type to reduce the overall chronic response are unlikely to be successful. The differences between our findings and typical *in vivo* responses indicate the importance of components other than native brain cells in the progression of the reactive tissue response. Our findings additionally point to a viable alternative hypothesis regarding neuronal density depletion following microelectrode implantation in the brain.

## Conflict of interest statement

The authors declare that the research was conducted in the absence of any commercial or financial relationships that could be construed as a potential conflict of interest.
